# Deubiquitinase inhibitor PR-619 reduces Smad4 expression and suppresses renal fibrosis in mice with unilateral ureteral obstruction

**DOI:** 10.1371/journal.pone.0202409

**Published:** 2018-08-16

**Authors:** Kotaro Soji, Shigehiro Doi, Ayumu Nakashima, Kensuke Sasaki, Toshiki Doi, Takao Masaki

**Affiliations:** Department of Nephrology, Hiroshima University Hospital, Hiroshima, Japan; Universidade de Sao Paulo, BRAZIL

## Abstract

Deubiquitinating enzymes (DUBs) remove ubiquitin from their substrates and, together with ubiquitin ligases, play an important role in the regulation of protein expression. Although transforming growth factor (TGF)-β1-Smad signaling is a central pathway of renal fibrosis, the role of DUBs in the expression of TGF-β receptors and Smads during the development of renal fibrosis remains unknown. In this study, we investigated whether PR-619, a pan-DUB inhibitor, suppresses fibrosis in mice with unilateral ureteral obstruction (UUO) and TGF-β1-stimulated normal rat kidney (NRK)-49F cells, a rat renal fibroblast cell line. Either the vehicle (dimethyl sulfoxide) or PR-619 (100 μg) was intraperitoneally administered to mice after UUO induction once a day for 7 days. Administration of PR-619 attenuated renal fibrosis with downregulation of mesenchymal markers, extracellular matrix proteins, matrix metalloproteinases, apoptosis, macrophage infiltration, and the TGF-β1 mRNA level in UUO mice. Although type I TGF-β receptor (TGF-βRI), Smad2, Smad3, and Smad4 protein expression levels were markedly increased in mice with UUO, administration of PR-619 suppressed only Smad4 expression but not TGF-βRI, Smad2, or Smad3 expression. PR-619 also had an inhibitory effect on TGF-β1-induced α-smooth muscle actin expression and reduced Smad4 levels in NRK-49F cells. Our results indicate that PR-619 ameliorates renal fibrosis, which is accompanied by the reduction of Smad4 expression.

## Introduction

Chronic kidney disease (CKD) is estimated to affect 13%–15% of the population in developed countries and is therefore recognized as a major public health problem globally with a substantial socioeconomic burden [1, 2]. Regardless of the underlying disease leading to CKD, renal fibrosis is a common pathological feature characterized by the proliferation of interstitial myofibroblasts, accumulation of excessive extracellular matrix (ECM), loss of the normal kidney structure with increased apoptotic cells, and infiltration of inflammatory cells [3, 4]. Renal fibrosis also has a strong correlation with the deterioration of renal function and the incidence of end-stage kidney disease in the clinical setting [5, 6]. However, therapeutic strategies for renal fibrosis have yet to be established.

Although several cytokines are involved in the development of renal fibrosis, transforming growth factor (TGF)-β1 has been identified as a key mediator in this process [7, 8]. TGF-β1 is the most abundant isoform of the TGF-β family and is secreted by all types of intrinsic renal cells and infiltrated inflammatory cells [9]. TGF-β1 binds to the type II TGF-β receptor (TGF-βRII) that recruits the type I TGF-β receptor (TGF-βRI). Subsequently, Smad2/3 are phosphorylated and form an oligomeric complex with Smad4. Finally, the Smad complex translocates into the nucleus to regulate transcription of profibrotic target genes [9, 10]. To suppress TGF-β1 signaling, downregulation of their expression could be a candidate therapeutic strategy for renal fibrosis.

Ubiquitination is a post-translational modification that targets a protein for degradation by the proteasome [11]. Previous studies have implicated ubiquitin-mediated degradation in numerous physiological processes including signal transduction, cell cycle regulation, protein trafficking, and DNA damage and repair [12–14]. Notably, ubiquitination is a reversible modification, and deubiquitinating enzymes (DUBs) preserve protein expression through the removal of ubiquitin from their substrates. Recently, DUBs have emerged as potential targets for pharmacological intervention of various diseases including neurological disorders, infectious diseases, and cancer [15–17]. However, the role of DUBs during the induction and progression of renal fibrosis remains to be elucidated.

Theoretically, inhibition of DUBs maintains ubiquitination, leading to the promotion of protein degradation. Regarding TGF-β1 signaling, previous studies in the field of cancer research have demonstrated that several DUBs enhance TGF-β1-Smad signaling by maintaining the expression of TGF-β1 signaling-related molecules such as TGF-βRI, Smad2/3, and Smad4 [18–21]. These findings led us to hypothesize that DUB inhibitors attenuate renal fibrosis by disrupting TGF-β1 signaling. To test this hypothesis, we investigated whether PR-619, a pan-DUB inhibitor, altered expression of profibrotic markers and TGF-β1-Smad signaling molecules in a mouse model of renal fibrosis and TGF-β1-stimulated renal cells.

In this study, we demonstrate that PR-619 suppresses renal fibrosis and reduces Smad4 expression but not the expression of TGF-β receptors, Smad2, or Smad3, in mice with unilateral ureteral obstruction (UUO), a well-established mouse model of renal fibrosis. We also show that PR-619 inhibits TGF-β1-induced fibrotic changes in normal rat kidney (NRK)-49F cells, a rat renal fibroblast cell line. Although TGF-β1 does not directly upregulate Smad4 expression, PR-619 reduces Smad4 expression in stimulated NRK-49F cells. These results suggest that PR-619 reduces Smad4 expression and thus attenuates renal fibrosis.

## Materials and methods

### Animals

Eight-week-old male C57BL/6J mice, weighing 22–25 g, were purchased from Charles River Laboratories Japan (Yokohama, Japan). The mice had free access to laboratory chow and tap water and were housed in a light- and temperature-controlled room at the Institute of Laboratory Animal Science of Hiroshima University (Hiroshima, Japan). The Institutional Animal Care and Use Committee of Hiroshima University approved all experimental protocols (permit number: A16-47). All experiments were performed in accordance with the National Institutes of Health (NIH) Guidelines on the Use of Laboratory Animals. The mice were randomly assigned to either control (n = 5), UUO with PR-619 treatment (n = 5), or UUO with vehicle treatment (n = 5) groups. UUO was performed by double ligation of the left ureter under general anesthesia (medetomidine, midazolam, and butorphanol). The control group of mice was subjected to a sham operation that was identical to the operation performed in mice with UUO except without ureter ligation. Mice were euthanized on day 7 after UUO, and kidneys were harvested. To investigate the effect of PR-619 on renal fibrosis after UUO, either 100 μg PR-619 (LifeSensors, Malvern, PA, USA) in 10 μL dimethyl sulfoxide (DMSO) or an equal volume of vehicle was intraperitoneally administered immediately after the UUO operation. We did not perform any post-operative care such as analgesia or anesthesia. The treatment was repeated every 24 h until the mice were euthanized 7 days after UUO surgery. We monitored the operated mice once a day, and no adverse effects, including death, were observed in all experimental mice.

### Western blotting

Sample collection and immunoblotting were performed as described previously [22]. The primary antibodies used in this study were a mouse monoclonal anti-α-smooth muscle actin (α-SMA) antibody (A2547, Sigma-Aldrich, St. Louis, MO, USA), mouse monoclonal anti-fibronectin antibody (F6140, Sigma-Aldrich), mouse monoclonal anti-glyceraldehyde 3-phosphate dehydrogenase (GAPDH) antibody (G8795, Sigma-Aldrich), mouse monoclonal anti-β-actin antibody (A5316, Sigma-Aldrich), rabbit monoclonal anti-Smad2 antibody (#5339, Cell Signaling Technology, Danvers, MA, USA), rabbit monoclonal anti-Smad3 antibody (#9523, Cell Signaling Technology), rabbit monoclonal anti-Smad4 antibody (#38454, Cell Signaling Technology), rabbit polyclonal anti-TGF-β receptor I antibody (ab31013, Abcam, Cambridge, UK), rabbit polyclonal anti-TGF-β receptor II antibody (ab186838, Abcam), rabbit polyclonal anti-matrix metalloproteinase (MMP) 2 antibody (ab37150, Abcam), and rabbit polyclonal anti-MMP9 antibody (ab38898, Abcam). Secondary antibodies used in this study were horseradish peroxidase-conjugated goat anti-rabbit immunoglobulin G (P0448, DAKO, Glostrup, Denmark) and anti-mouse immunoglobulin G (P0447, DAKO). Signals were visualized by SuperSignal West Dura and Pico systems (Thermo Fisher, Rockford, IL, USA). The intensity of each band was quantified using ImageJ software (version 1.46r; NIH, Bethesda, MD, USA).

### Histological analysis and immunohistochemistry

Histological and immunohistochemical staining were performed as described previously [23]. Hematoxylin-eosin (HE) staining was performed to evaluate the degree of cellularity. Interstitial cells were counted using ImageJ software by examination of predetermined ×200 power fields of the cortex (five fields). Masson’s trichrome (MT) staining was performed to assess the severity of tubulointerstitial fibrosis. The fibrotic area (labeled blue) was quantified using Lumina Vision 2.20 (Mitani, Osaka, Japan) by examination of a predetermined ×40 power field. Apoptotic cells were detected in paraffin-embedded sections using the DeadEnd Colorimetric TUNEL (terminal deoxynucleotidyl transferase dUTP nick end labeling) System (Promega, Madison, WI, USA), according to the manufacturer’s protocol. The following primary antibodies were used for immunohistochemical staining: mouse monoclonal anti-αSMA antibody (A2547, Sigma-Aldrich), mouse monoclonal anti-fibronectin antibody (F6140, Sigma-Aldrich), rabbit polyclonal anti-S100A4 (also known as fibroblast-specific protein-1; FSP-1) antibody (ab27957, Abcam), rabbit polyclonal anti-collagen 1 antibody (ab34710, Abcam), rabbit polyclonal anti-collagen 3 antibody (ab7778, Abcam), and mouse monoclonal anti-CD68 antibody (ab955, Abcam). Positive areas for these antibodies, except TUNEL staining, were quantified using color limits for the positive pixels defined in ImageJ software by examination of a predetermined ×40 power field of the cortex. The number of TUNEL-positive cells was counted in a predetermined ×200 power fields of the cortex (five fields).

### Cell culture

The NRK-49F cell line was obtained from the American Type Culture Collection (Manassas, VA, USA). The cells were maintained in Dulbecco’s modified Eagle’s medium (DMEM) (Nacalai Tesque, Kyoto, Japan) containing 5% fetal bovine serum (FBS) (Nichirei Bio Science, Tokyo, Japan) and penicillin/streptomycin (Nacalai Tesque). To test the effects of PR-619 on the cultured cell line, 3 μmol/L PR-619 (LifeSensors) was applied to subconfluent cells for 60 min before TGF-β1 stimulation. Cells were then exposed to TGF-β1 (10 ng/mL) in the presence or absence of PR-619 for 24 h. Whole cell lysates were prepared and subjected to western blot analysis.

### RNA extraction and quantitative real-time RT-PCR (qRT-PCR)

RNA extraction and qRT-PCR were performed as described previously [24]. Briefly, qRT-PCR was performed using the ABI 7500 fast real-time PCR system (Applied Biosystems, Foster City, CA, USA). The specific oligonucleotide primers and probes for α-SMA (assay ID: Mm00725412_s1), fibronectin (assay ID: Mm01256744_m1), collagen 1 (assay ID: Mm00801666_g1), collagen 3 (assay ID: Mm01254476_m1), MMP2 (assay ID: Mm00439498_m1), MMP9 (assay ID: Mm00442991_m1), TGF-β1 (assay ID: Mm00441727_m1), and GAPDH (assay ID: Mm99999915_g1) as an internal control were all TaqMan Gene Expression Assays (Applied Biosystems).

### Statistical analysis

Results are expressed as the mean ± standard deviation (SD). Statistical analysis was performed using analysis of variance (ANOVA), followed by Tukey’s post hoc test. A value of *P* < 0.05 was considered to indicate a statistically significant difference.

## Results

### PR-619 improves renal histopathological changes in mice with UUO

**Fig 1:**
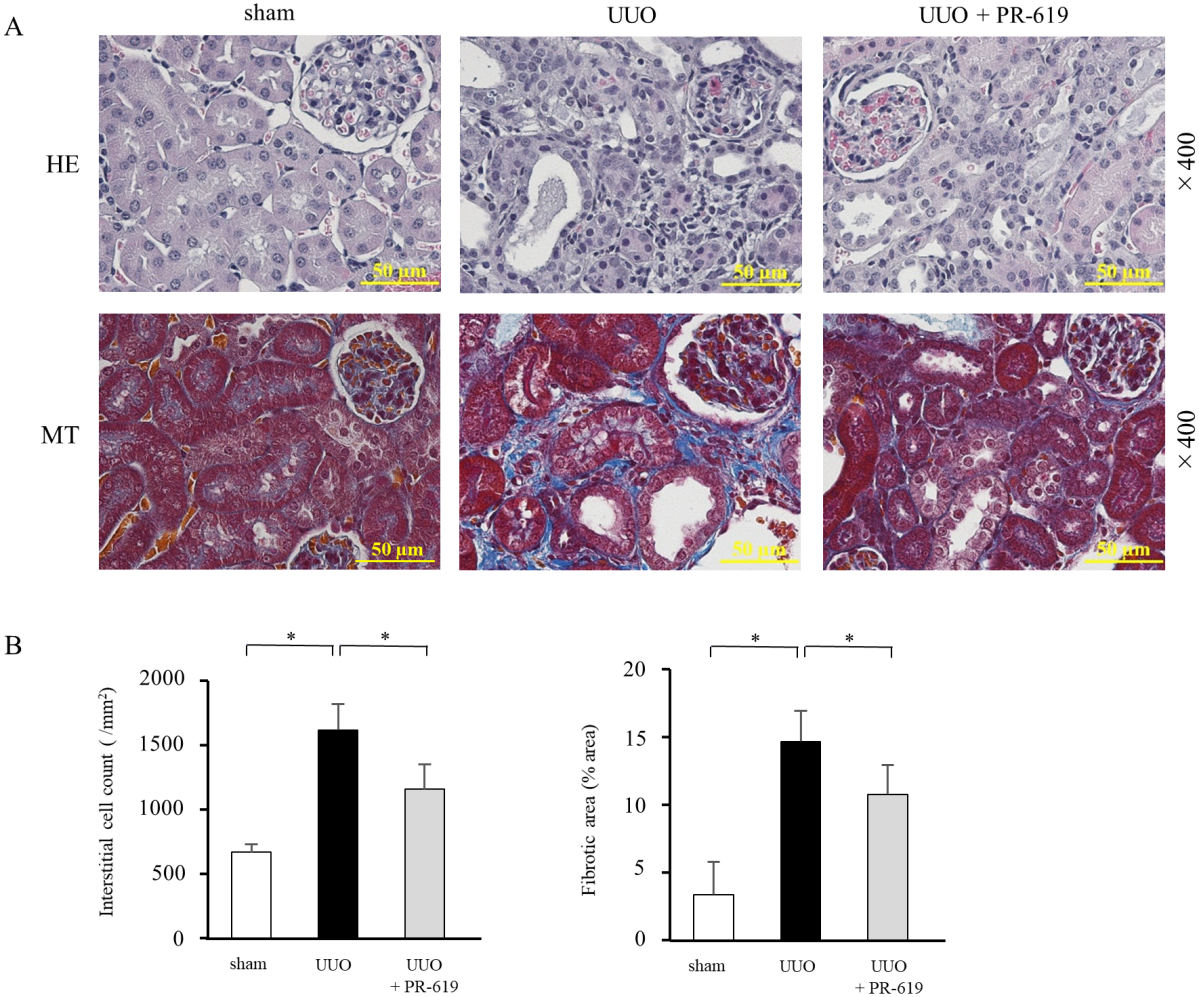
PR-619 improves renal histopathological changes in mice with UUO. Mice were treated daily with 100 μg PR-619 in 10 μL DMSO or an equal volume of vehicle by intraperitoneal injection. Paraffin-embedded sections of UUO kidneys were stained with HE and MT. (A) Representative images of HE (upper panel) and MT (lower panel) staining of kidney sections from sham-operated mice and mice with UUO treated with or without PR-619. (B) Quantification of the interstitial cell density (left panel) and interstitial fibrosis (right panel). HE staining was used to assess the interstitial cell count. MT staining was used to detect connective tissue as indicated by the blue staining. Values are expressed as the mean ± SD. Statistical analysis was performed using ANOVA followed by Tukey’s post hoc test. *P < 0.05, n = 5 mice per group. DMSO, dimethyl sulfoxide; UUO, unilateral ureteral obstruction; HE, hematoxylin-eosin; MT, Masson’s trichrome; SD, standard deviation; ANOVA, analysis of variance.

UUO induces renal fibrosis characterized by increased cellularity and ECM accumulation in the interstitial space [25, 26]. To investigate the effect of DUB inhibition on UUO-induced renal fibrosis, we first performed histological examinations. HE staining was used to assess changes in interstitial cell density, and MT staining was used to analyze interstitial fibrosis. Administration of PR-619 significantly attenuated interstitial cell infiltration, which was accompanied by a reduction of the interstitial fibrotic area in mice with UUO (Fig 1A and 1B).

### PR-619 ameliorates expression of mesenchymal markers in the kidneys of mice with UUO

**Fig 2:**
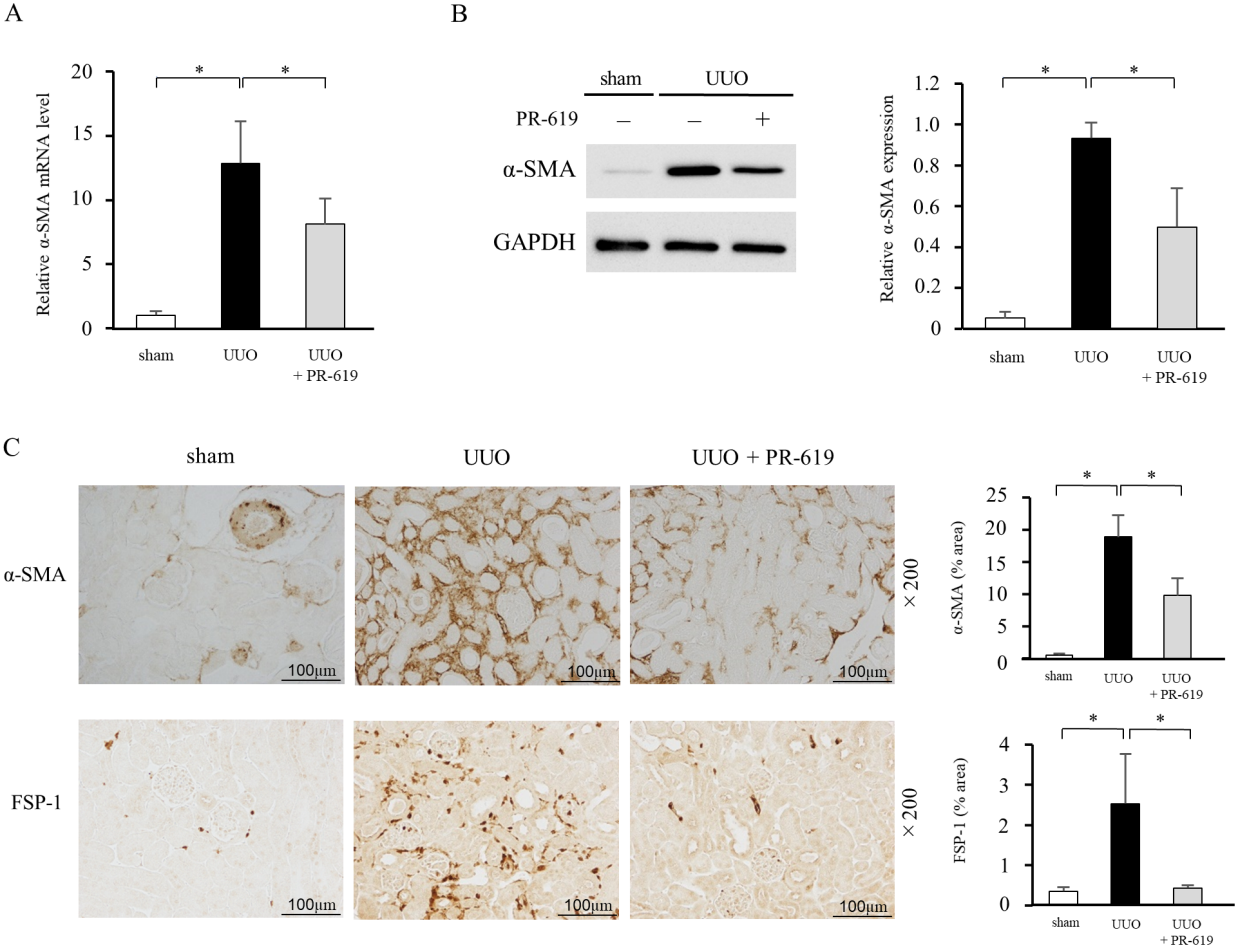
PR-619 ameliorates expression of mesenchymal markers in the kidneys of mice with UUO. Kidney samples were collected from mice with UUO after vehicle or PR-619 treatment and examined for expression of α-SMA and FSP-1 as mesenchymal markers. (A) α-SMA mRNA levels were determined by qRT-PCR in mice with UUO with or without PR-619 administration. GAPDH was used as an internal control. (B) Typical western blot analysis demonstrating the level of α-SMA protein expression. The graph shows the expression level quantified by densitometry and normalized to GAPDH. (C) Representative images showing immunostaining for α-SMA and FSP-1. Quantification is shown in the right panel. Values are expressed as the mean ± SD. Statistical analysis was performed using ANOVA followed by Tukey’s post hoc test. *P < 0.05, n = 5 mice per group. UUO, unilateral ureteral obstruction; α-SMA, α-smooth muscle actin; FSP-1, fibroblast-specific protein-1; qRT-PCR, quantitative real-time RT-PCR; GAPDH, glyceraldehyde 3-phosphate dehydrogenase; SD, standard deviation; ANOVA, analysis of variance.

Acquisition of the mesenchymal phenotype is an important feature in the progression of renal fibrosis, which contributes to the production of ECM proteins. We therefore examined the expression of α-SMA and FSP-1 as mesenchymal markers. qRT-PCR and western blot analysis showed that α-SMA was hardly detectable in the kidney tissue of sham-operated mice, and that UUO resulted in a marked increase in α-SMA expression. Notably, administration of PR-619 attenuated α-SMA expression in mice with UUO (Fig 2A and 2B). Immunohistochemical staining also revealed that UUO-induced expression of α-SMA and FSP-1 in the kidney tissue was reduced by PR-619 treatment (Fig 2C).

### PR-619 suppresses UUO-induced ECM deposition

**Fig 3:**
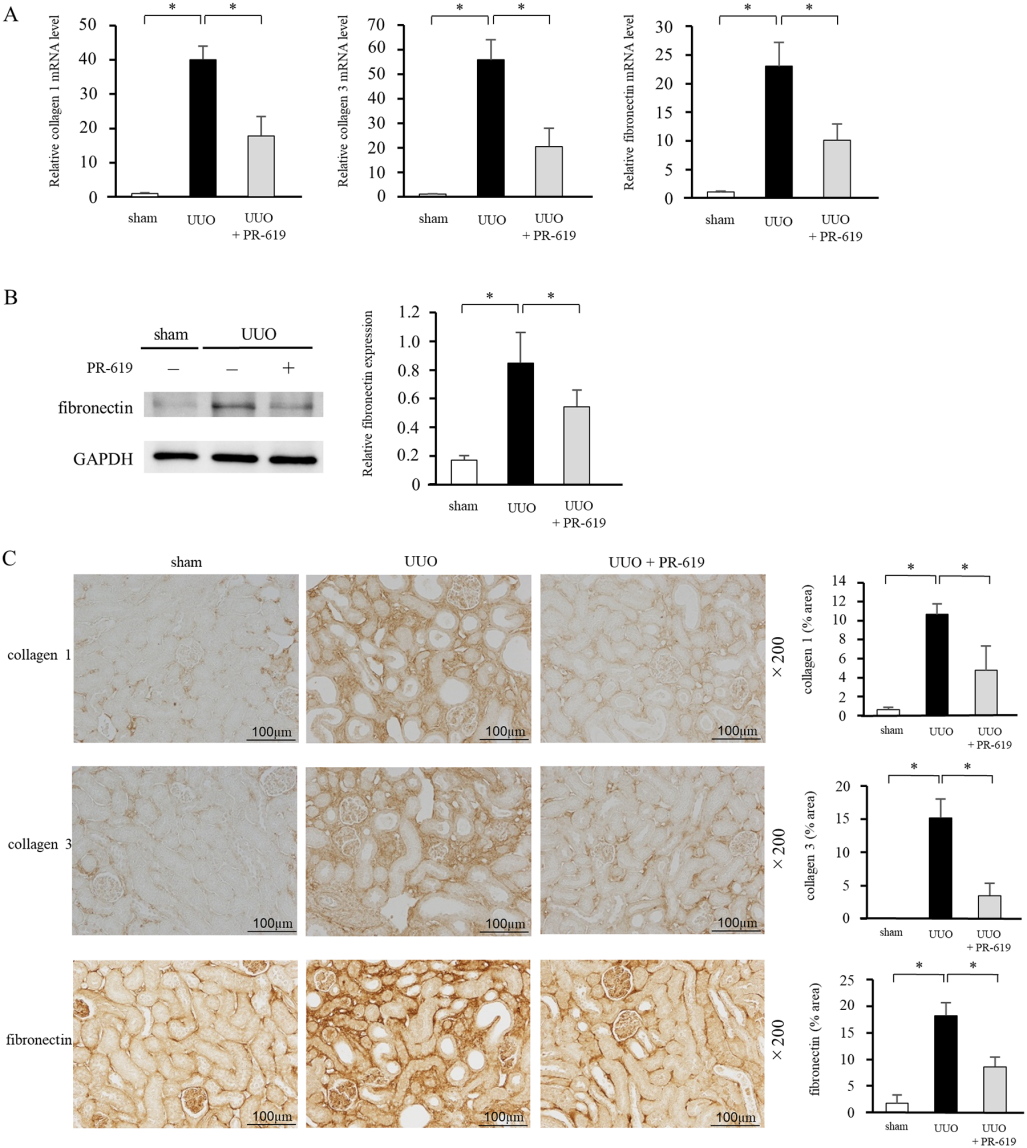
PR-619 suppresses UUO-induced ECM deposition. The same samples in Fig 2 were used to examine collagen 1, collagen 3, and fibronectin as ECM proteins. (A) Collagen 1, collagen 3, and fibronectin mRNA levels were determined by qRT-PCR in mice with UUO with or without PR-619 administration. GAPDH was used as an internal control. (B) Typical western blot analysis demonstrating the level of fibronectin protein expression. The graph shows the expression level quantified by densitometry and normalized to GAPDH. (C) Representative images showing immunostaining for collagen 1, collagen 3, and fibronectin. Quantification is shown in the right panel. Values are expressed as the mean ± SD. Statistical analysis was performed using ANOVA followed by Tukey’s post hoc test. *P < 0.05, n = 5 mice per group. ECM, extracellular matrix; qRT-PCR, quantitative real-time RT-PCR; UUO, unilateral ureteral obstruction; GAPDH, glyceraldehyde 3-phosphate dehydrogenase; SD, standard deviation; ANOVA, analysis of variance.

Because ECM accumulation is a central process of tubulointerstitial fibrosis, we examined expression of collagen 1, collagen 3, and fibronectin as major ECM proteins. qRT-PCR and immunohistochemical analysis showed that the levels of collagen 1, collagen 3, and fibronectin were markedly increased in UUO mice compared with sham-operated mice (Fig 3A and 3C). Western blot analysis confirmed that the protein expression of fibronectin was enhanced in UUO mice (Fig 3B). Furthermore, PR-619 treatment reduced UUO-induced ECM expression levels.

### PR-619 reduces UUO-induced MMP expression, apoptosis, and inflammation

**Fig 4:**
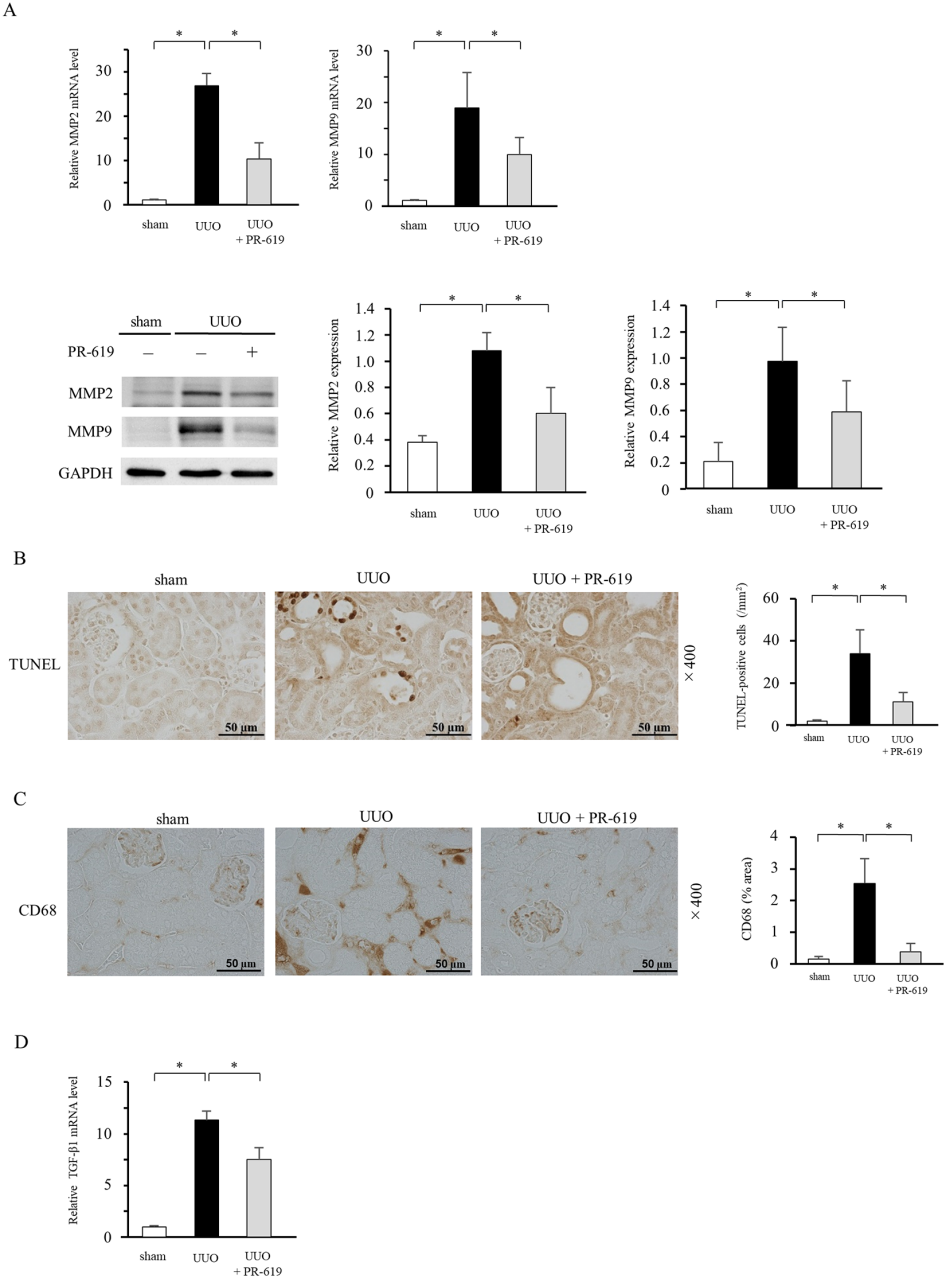
PR-619 reduces UUO-induced MMP expression, apoptosis, and inflammation. The same samples in Figs 2 and 3 were used to investigate UUO-induced tissue damage including expression of MMPs, TUNEL-positive apoptotic cell death, CD68-positive macrophage infiltration, and TGF-β1 mRNA levels. (A) MMP2 and MMP9 mRNA levels were determined by qRT-PCR in mice with UUO with or without PR-619 administration. GAPDH was used as an internal control (upper panel). Typical western blot analysis demonstrating the levels of MMP2 and MMP9 protein expression. The graphs show the expression level quantified by densitometry and normalized to GAPDH (lower panel). (B) Representative photomicrograph of TUNEL staining in kidney sections. Quantification of TUNEL-positive cells is shown in the right panel. (C) Representative images showing CD68 immunostaining. Quantification is shown in the right panel. (D) TGF-β1 mRNA levels were determined by qRT-PCR. GAPDH was used as an internal control. Values are expressed as the mean ± SD. Statistical analysis was performed using ANOVA followed by Tukey’s post hoc test. *P < 0.05, n = 5 mice per group. UUO, unilateral ureteral obstruction; MMP, matrix metalloproteinase; TUNEL, terminal deoxynucleotidyl transferase dUTP nick end labeling; TGF-β1, transforming growth factor-β1; qRT-PCR, quantitative real-time RT-PCR; GAPDH, glyceraldehyde 3-phosphate dehydrogenase; SD, standard deviation; ANOVA, analysis of variance.

To further assess the effect of PR-619 on UUO-induced renal damage, we examined expression of MMPs, the degree of apoptosis, and macrophage infiltration. Considering that TGF-β1 plays a central role in the development of fibrosis, we also investigated TGF-β1 mRNA levels in UUO mice with or without PR-619 treatment. qRT-PCR and western blot analysis revealed increases of MMP2 and MMP9 expression in UUO mice, but PR-619 significantly mitigated their upregulation (Fig 4A). TUNEL-positive cells were significantly increased in UUO mice treated with the vehicle only, but they were decreased in UUO mice with PR-619 administration (Fig 4B). Similarly, PR-619 suppressed infiltration of CD68 (macrophage marker)-positive cells in UUO mice (Fig 4C). Expression levels of TGF-β1 mRNA were upregulated in UUO mice treated with the vehicle only, but they were downregulated by PR-619 administration (Fig 4D).

### PR-619 decreases Smad4 expression in UUO mice

**Fig 5:**
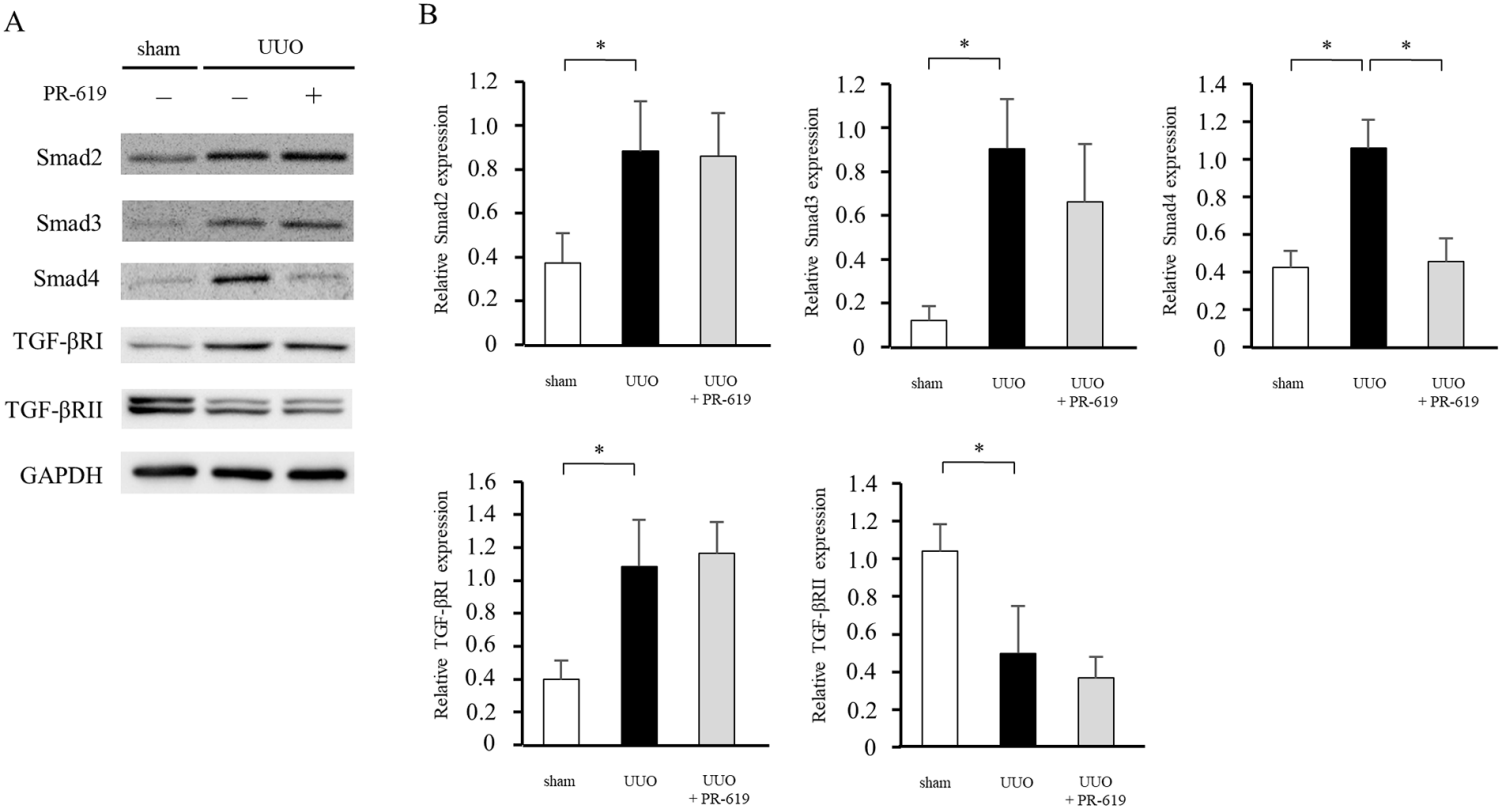
PR-619 decreases Smad4 expression in UUO mice. The same protein lysates in Figs 2–4 were used to examine expression of TGF-β1-Smad signaling molecules. (A) Typical western blot analysis demonstrating the total levels of Smad2, Smad3, Smad4, TGF-βRI, and TGF-βRII protein expression in mice with UUO with or without PR-619 administration. (B) Quantification of the expression of these proteins on a relative scale. The band intensities were normalized to GAPDH. Values are expressed as the mean ± SD. Statistical analysis was performed using ANOVA followed by Tukey’s post hoc test. *P < 0.05, n = 5 mice per group. TGF-β1, transforming growth factor-β1; TGF-βRI, Type I TGF-β receptor; TGF-βRII, Type II TGF-β receptor; UUO, unilateral ureteral obstruction; GAPDH, glyceraldehyde 3-phosphate dehydrogenase; SD, standard deviation; ANOVA, analysis of variance.

TGF-β1 expression plays a key role in the development of renal fibrosis in mice with UUO [27, 28]. To investigate the role of DUBs in UUO-induced fibrosis, we evaluated TGF-β1 signaling molecules, namely TGF-β receptors, Smad2, Smad3, and Smad4, in PR-619-treated mice. As shown in Fig 5, compared with sham controls, TGF-βRI, Smad2, Smad3, and Smad4 protein expression levels were markedly increased in UUO mice, while TGF-βRII protein expression level was decreased. PR-619 administration suppressed Smad4 protein expression but not TGF-β receptor, Smad2, or Smad3 expression in UUO mice.

### PR-619 attenuates TGF-β1-induced myofibroblastic changes and reduces Smad4 expression in NRK-49F cells

**Fig 6:**
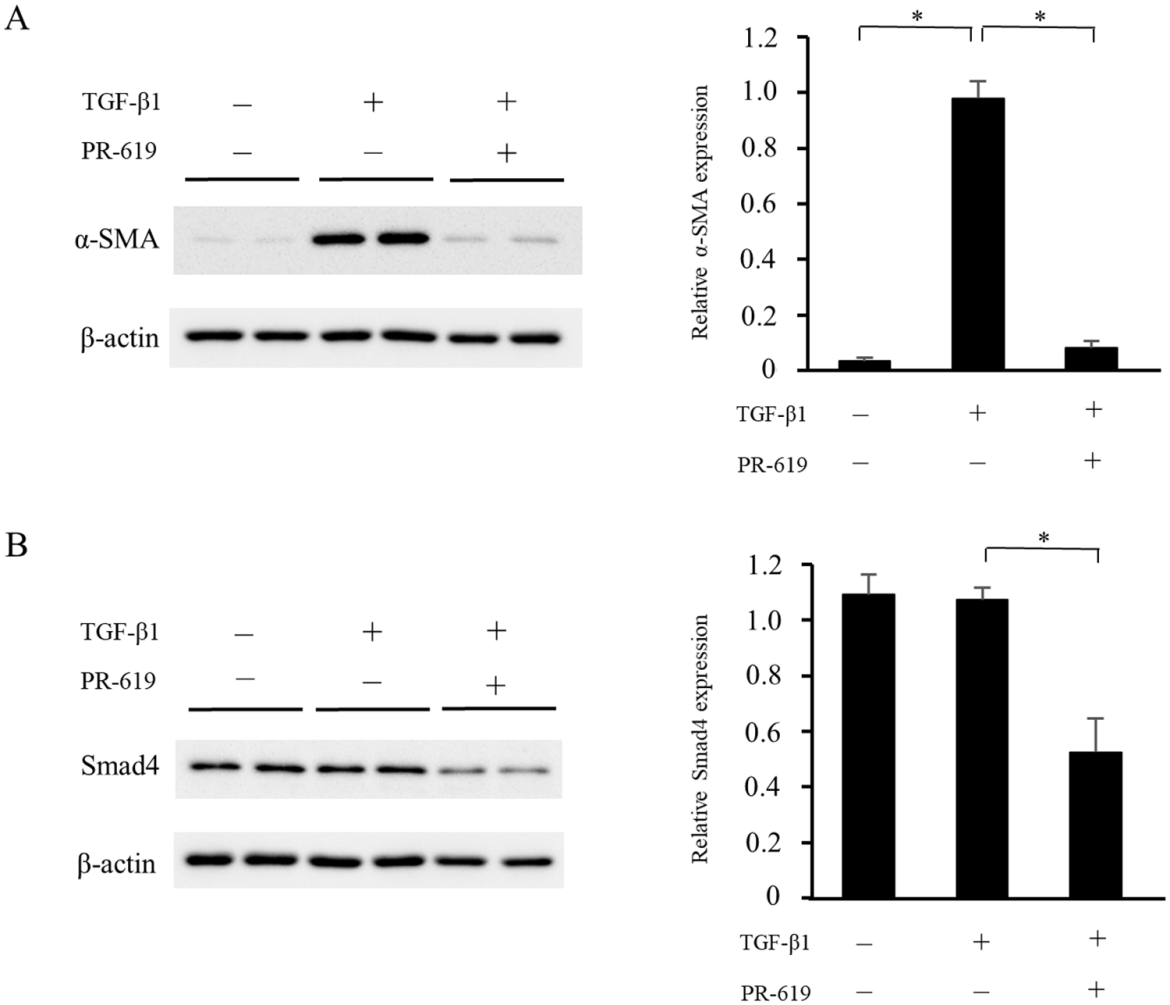
PR-619 attenuates TGF-β1-induced myofibroblastic changes and reduces Smad4 expression in NRK-49F cells. NRK-49F renal interstitial fibroblasts were incubated with PR-619 (3 μmol/L) for 60 min and then stimulated with TGF-β1 (10 ng/mL) for 24 h. Cell lysates were subjected to western blot analysis using antibodies against α-SMA or Smad4. Typical western blot analysis demonstrating the expression levels of (A) α-SMA and (B) Smad4. Graphs show the expression levels quantified by densitometry and normalized to β-actin. Values are expressed as the mean ± SD. Statistical analysis was performed using ANOVA followed by Tukey’s post hoc test. *P < 0.05, n = 5 samples per group. NRK-49F, normal rat kidney-49F; TGF-β1, transforming growth factor-β1; α-SMA, α-smooth muscle actin; SD, standard deviation; ANOVA; analysis of variance.

Resident renal fibroblasts are a major source of myofibroblasts in the fibrotic kidney [29], and TGF-β1-induced myofibroblastic changes play a crucial role in the development of renal fibrosis. We examined the effects of DUB inhibition on fibrotic changes in TGF-β1-stimulated NRK-49F cells treated with or without PR-619 for 24 h. As shown in Fig 6, TGF-β1-induced α-SMA expression was suppressed by PR-619 treatment. Although protein expression of Smad4 was not directly upregulated by TGF-β1 stimulation, PR-619 administration significantly reduced Smad4 expression.

## Discussion

In this study, we show that the pan-DUB inhibitor PR-619 reduces expression of mesenchymal markers, deposition of ECM proteins, expression of MMP2 and MMP9, the degree of apoptosis, macrophage infiltration, and the TGF-β1 mRNA level, and attenuates renal fibrosis. We also show that the beneficial effect of PR-619 treatment is accompanied by a reduction in Smad4 expression but not TGF-β receptor, Smad2, or Smad3 expression. In the *in vitro* experiments, PR-619 treatment inhibited TGF-β1-induced α-SMA expression and reduced Smad4 expression, although TGF-β1 did not induce Smad4 expression. These results indicate that pharmacological inhibition of DUBs ameliorates renal fibrosis concomitantly with the reduction of Smad4 expression.

We showed that the deubiquitinase inhibitor PR-619 suppressed renal fibrosis in mice with UUO. Ubiquitin ligases contribute to protein degradation in conjunction with the proteasome, whereas DUBs maintain protein expression. Therefore, the beneficial effect of PR-619 may be attributable to the promotion of protein degradation. Regarding renal fibrosis, previous studies have demonstrated that autophagy-induced protein degradation contributes to suppression of renal fibrosis by UUO [30, 31], suggesting that downregulation of protein expression is a therapeutic strategy during the development of renal fibrosis. However, autophagy is a bulk protein degradation system, whereas the ubiquitin-proteasome system performs more specific regulation of protein expression than autophagy [12, 32, 33]. Therefore, intervention of the ubiquitin-proteasome system is a more attractive therapeutic strategy for renal fibrosis.

TGF-β1 is a key cytokine that contributes to transdifferentiation of renal cells to myofibroblasts, leading to the production of ECM proteins in various rodent models of renal fibrosis [7, 27, 34, 35]. Although TGF-β1 stimulates several pathways, multiple studies have identified TGF-β1-Smad signaling as the main pathway in renal fibrosis [9, 36]. In this study, we showed that PR-619 inhibited Smad4 expression without changes in expression of other molecules associated with TGF-β1-Smad signaling. As a component of the heteromeric complex formed with phosphorylated Smad2/3, Smad4 plays a crucial role in mediating signal transduction of TGF-β1 [9, 10]. Notably, Meng et al. reported that specific deletion of Smad4 from renal tubular epithelial cells ameliorates UUO-induced renal fibrosis by suppressing Smad3 responsive promoter activity and decreasing the binding of Smad3 to its target genes independent of its phosphorylation and nuclear translocation [37]. These findings suggest that the reduction of Smad4 contributed to the anti-fibrotic effect of PR-619 in UUO-induced renal fibrosis.

In this study, we showed that PR-619 also suppressed MMP2, MMP9, apoptosis, and inflammation. In addition to production of ECM protein, previous studies have shown that TGF-β1-Smad signaling directly contributes to tissue damage during the development of renal fibrosis, including MMP-mediated protein degradation and apoptosis [38–40]. Despite the established proinflammatory role, TGF-β1 also possesses anti-inflammatory effects [41]. However, a previous study has reported that genetic knockout of Smad3 attenuates not only renal fibrosis but also macrophage infiltration in UUO mice [42]. Interestingly, a recent study found that TGF-β1-Smad signaling promotes macrophage-to-myofibroblast transition during the progression of fibrosis [43]. These findings suggest that anti-fibrotic effects of PR-619 also result from the reduction of TGF-β1-induced tissue damage and inflammation-mediated fibrogenesis.

We have demonstrated that PR-619 suppressed UUO-induced upregulation of Smad4 but not TGF-β receptors, Smad2, or Smad3. A previous study suggested that ubiquitin-specific peptidase 9, X-linked (USP9X) is possibly responsible for Smad4 expression [21]. USP9X has been identified as a DUB, and multiple substrates of USP9X have been identified, including Smad4 [44]. However, we did not observe an inhibitory effect of USP9X siRNA on Smad4 expression in NRK-49F cells (S1 Fig). In addition, even though WP1130 is an inhibitor of USP9X [45, 46], administration of WP1130 did not suppress renal fibrosis or Smad4 expression in mice with UUO (S2 Fig). Moreover, although Smad4 expression was upregulated in UUO mice, TGF-β1 did not directly induce Smad4 expression in NRK-49 cells. Therefore, further studies are needed to clarify the precise mechanism underlying Smad4 expression and its degradation.

In summary, we provide evidence that PR-619, a pan-DUB inhibitor, suppresses fibrosis in the kidneys of mice with UUO and in a rat renal fibroblast cell line stimulated with TGF-β1. We also show that PR-619 suppresses Smad4 expression both *in vivo* and *in vitro*. Although we could not identify the specific DUB responsible for regulating Smad4 expression, our data suggest that inhibition of DUBs might be a candidate therapeutic treatment to reduce Smad4 expression and prevent renal fibrosis in CKD.

## Supporting information

S1 Fig Knockdown of USP9X does not suppress Smad4 expression in NRK-49F cells. We investigated whether suppression of USP9X is responsible for Smad4 expression. NRK-49F cells were transfected with siRNA oligonucleotides targeting USP9X (siUSP9X) or negative control siRNA (siNeg). The cells were then exposed to TGF-β1 (10 ng/mL) for 24 h. Cell lysates were subjected to western blot analysis with anti-USP9X (Cell Signaling Technology), -Smad4, and -β-actin antibodies. Typical western blots demonstrating the expression levels of USP9X and Smad4 are shown in the upper panel. Quantification is shown in the lower panel. β-actin was used as an internal control. Values are expressed as the mean ± SD. Statistical analysis was performed using ANOVA followed by Tukey’s post hoc test. *P < 0.05, n = 5 samples per group. USP9X, ubiquitin-specific peptidase 9, X-linked; NRK-49F, normal rat kidney-49F; siRNA, small interfering RNA; TGF-β1, transforming growth factor-β1; SD, standard deviation; ANOVA, analysis of variance.
(DOCX)

S2 Fig WP1130 does not attenuate α-SMA or Smad4 expression in mice with UUO. We evaluated the effect of WP1130 on kidney fibrosis in UUO mice. After induction of UUO, either 40 μg WP1130 (LifeSensors) in 10 μL DMSO or an equal volume of vehicle was intraperitoneally administered once a day for 7 days, and then renal tissues were harvested. Typical western blots demonstrating the expression levels of α-SMA and Smad4 are shown in the left panel. Quantification is shown in the right panel. GAPDH was used as an internal control. Values are expressed as the mean ± SD. Statistical analysis was performed using ANOVA followed by Tukey’s post hoc test. *P < 0.05, n = 5 mice per group. UUO, unilateral ureteral obstruction; DMSO, dimethyl sulfoxide; α-SMA, α-smooth muscle actin; GAPDH, glyceraldehyde 3-phosphate dehydrogenase; SD, standard deviation; ANOVA, analysis of variance.
(DOCX)

S1 Table Calculate intensities for all blots. (PDF)
