# Supplementary material for: Deubiquitinase inhibitor PR-619 reduces Smad4 expression and suppresses renal fibrosis in mice with unilateral ureteral obstruction
Source: PLoS One. 2018 Aug 16;13(8):e0202409. doi: 10.1371/journal.pone.0202409 (PMC6095583; doi:10.1371/journal.pone.0202409)
Supplement: S1 Fig — We investigated whether suppression of USP9X is responsible for Smad4 expression. NRK-49F cells were transfected with siRNA oligonucleotides targeting USP9X (siUSP9X) or negative control siRNA (siNeg). The cells were then exposed to TGF-β1 (10 ng/mL) for 24 h. Cell lysates were subjected to western blot analysis with anti-USP9X (Cell Signaling Technology), -Smad4, and -β-actin antibodies. Typical western blots demonstrating the expression levels of USP9X and Smad4 are shown in the upper panel. Quantification is shown in the lower panel. β-actin was used as an internal control. Values are expressed as the mean ± SD. Statistical analysis was performed using ANOVA followed by Tukey’s post hoc test. *P < 0.05, n = 5 samples per group. USP9X, ubiquitin-specific peptidase 9, X-linked; NRK-49F, normal rat kidney-49F; siRNA, small interfering RNA; TGF-β1, transforming growth factor-β1; SD, standard deviation; ANOVA, analysis of variance. (DOCX) [file pone.0202409.s001.docx]

**
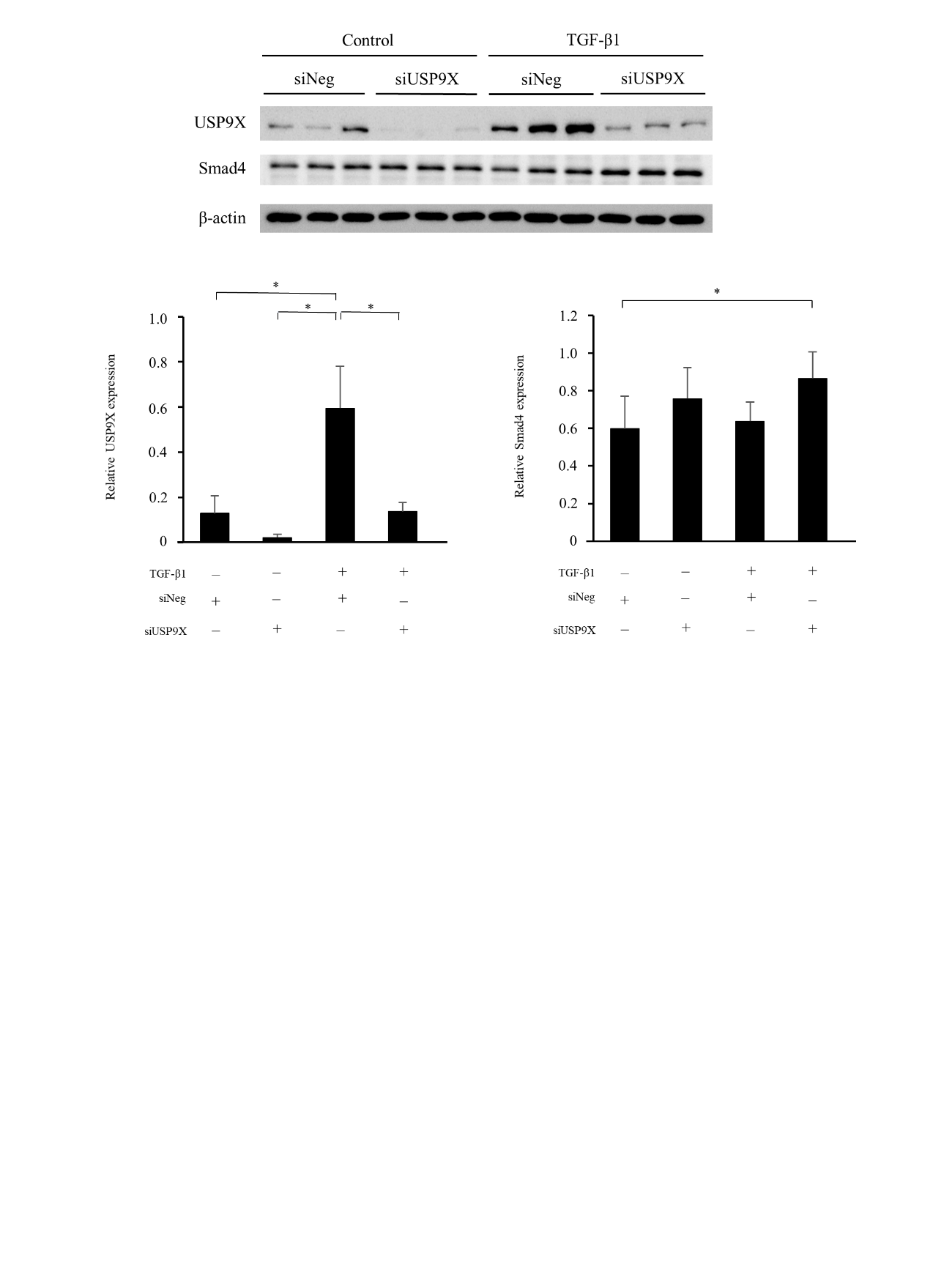
**

**S1 Fig. Knockdown of USP9X does not suppress Smad4 expression in NRK-49F cells.**

We investigated whether suppression of USP9X is responsible for Smad4 expression. NRK-49F cells were transfected with siRNA oligonucleotides targeting USP9X (siUSP9X) or negative control siRNA (siNeg). The cells were then exposed to TGF-β1 (10 ng/mL) for 24 h. Cell lysates were subjected to western blot analysis with anti-USP9X (Cell Signaling Technology), -Smad4, and -β-actin antibodies. Typical western blots demonstrating the expression levels of USP9X and Smad4 are shown in the upper panel. Quantification is shown in the lower panel. β-actin was used as an internal control. Values are expressed as the mean ± SD. Statistical analysis was performed using ANOVA followed by Tukey’s post hoc test. **P* < 0.05, n = 5 samples per group. USP9X, ubiquitin-specific peptidase 9, X-linked; NRK-49F, normal rat kidney-49F; siRNA, small interfering RNA; TGF-β1, transforming growth factor-β1; GAPDH, glyceraldehyde 3-phosphate dehydrogenase; SD, standard deviation; ANOVA, analysis of variance.
