# Supplementary material for: Deubiquitinase inhibitor PR-619 reduces Smad4 expression and suppresses renal fibrosis in mice with unilateral ureteral obstruction
Source: PLoS One. 2018 Aug 16;13(8):e0202409. doi: 10.1371/journal.pone.0202409 (PMC6095583; doi:10.1371/journal.pone.0202409)
Supplement: S2 Fig — We evaluated the effect of WP1130 on kidney fibrosis in UUO mice. After induction of UUO, either 40 μg WP1130 (LifeSensors) in 10 μL DMSO or an equal volume of vehicle was intraperitoneally administered once a day for 7 days, and then renal tissues were harvested. Typical western blots demonstrating the expression levels of α-SMA and Smad4 are shown in the left panel. Quantification is shown in the right panel. GAPDH was used as an internal control. Values are expressed as the mean ± SD. Statistical analysis was performed using ANOVA followed by Tukey’s post hoc test. *P < 0.05, n = 5 mice per group. UUO, unilateral ureteral obstruction; DMSO, dimethyl sulfoxide; α-SMA, α-smooth muscle actin; GAPDH, glyceraldehyde 3-phosphate dehydrogenase; SD, standard deviation; ANOVA, analysis of variance. (DOCX) [file pone.0202409.s002.docx]

**
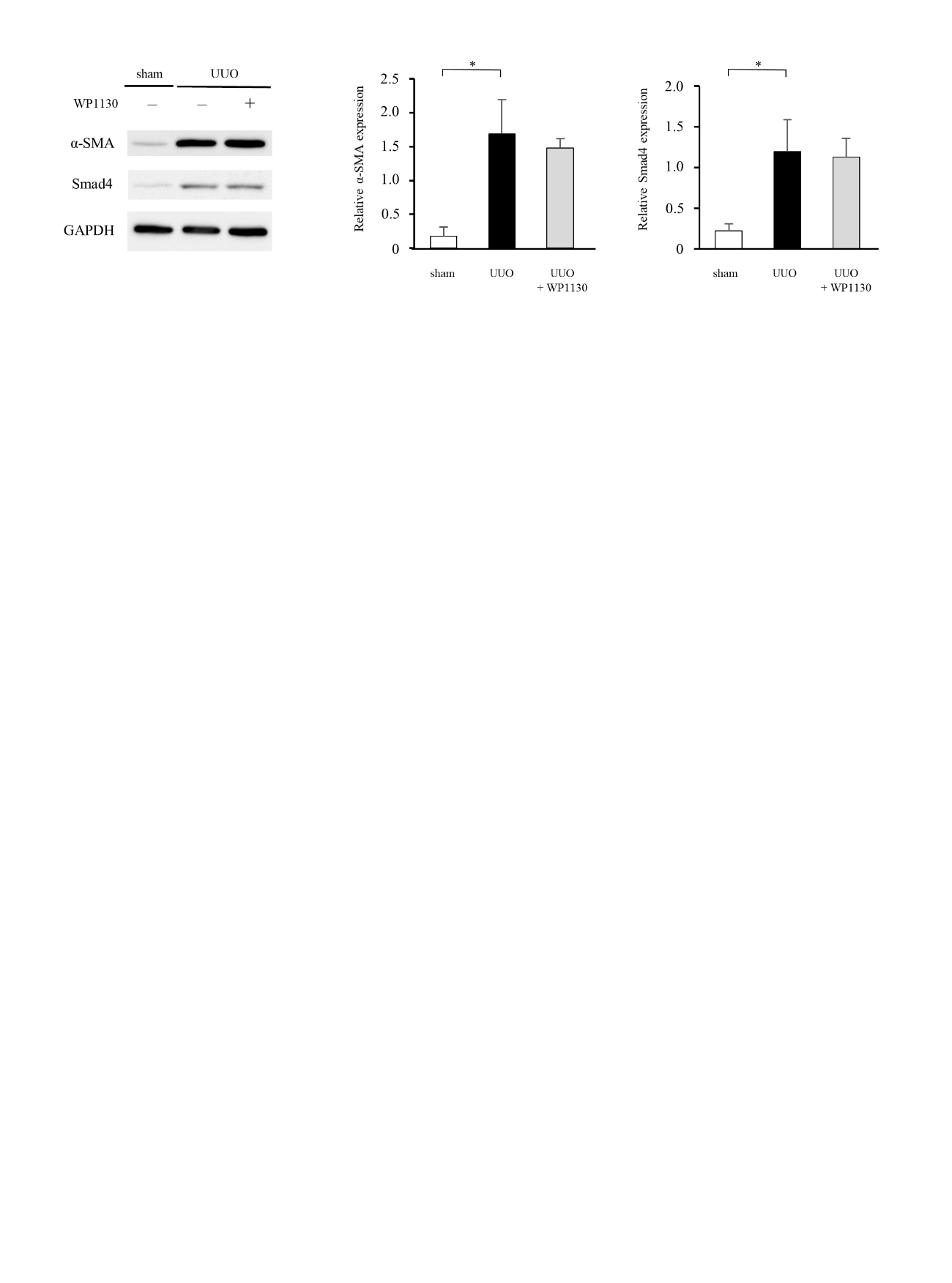
**

**S2 Fig. WP1130 does not attenuate α-SMA or Smad4 expression in mice with UUO.**

We evaluated the effect of WP1130 on kidney fibrosis in UUO mice. After induction of UUO, either 40 µg WP1130 (LifeSensors) in 10 µL DMSO or an equal volume of vehicle was intraperitoneally administered once a day for 7 days, and then renal tissues were harvested. Typical western blots demonstrating the expression levels of α-SMA and Smad4 are shown in the left panel. Quantification is shown in the right panel. GAPDH was used as an internal control. Values are expressed as the mean ± SD. Statistical analysis was performed using ANOVA followed by Tukey’s post hoc test. **P* < 0.05, n = 5 mice per group. UUO, unilateral ureteral obstruction; DMSO, dimethyl sulfoxide; α-SMA, α-smooth muscle actin; GAPDH, glyceraldehyde 3-phosphate dehydrogenase; SD, standard deviation; ANOVA, analysis of variance.
