# Supplementary material for: Deubiquitinase inhibitor PR-619 reduces Smad4 expression and suppresses renal fibrosis in mice with unilateral ureteral obstruction
Source: PLoS One. 2018 Aug 16;13(8):e0202409. doi: 10.1371/journal.pone.0202409 (PMC6095583; doi:10.1371/journal.pone.0202409)
Supplement: S1 Table — (PDF) [file pone.0202409.s003.pdf]

Figure2B

		α-SMA	GAPDH	α-SMA/GAPDH	mean	SD
sham	1	52.485	21888.719	0.00239781	0.051663745	0.032074661
	2	1409.698	20441.134	0.068963786		
	3	829.698	22483.962	0.03690177		
	4	1846.406	22706.962	0.081314533		
	5	1332.991	19391.548	0.068740825		
UUO	1	17451.134	18753.376	0.930559596	0.931763906	0.077771605
	2	17369.719	18636.255	0.932039136		
	3	20185.841	19151.548	1.054005713		
	4	17294.841	20580.255	0.84036087		
	5	17074.134	18932.255	0.901854217		
UUO + PR-619	1	7505.477	20533.376	0.365525718	0.4973585	0.188843128
	2	15161.305	19392.376	0.781817813		
	3	10366.891	18206.426	0.569408351		
	4	9494.598	20268.376	0.468443944		
	5	5157.77	17101.548	0.301596674		

Figure3B

		fibronectin	GAPDH	fibronectin/GAPDH	mean	SD
sham	1	2872.74	21986.548	0.130658983	0.169351609	0.031729032
	2	3212.225	17698.255	0.181499532		
	3	3402.17512	17250.841	0.197217928		
	4	3472.487672	17625.255	0.197017727		
	5	2128.472434	15163.962	0.140363873		
UUO	1	11777.761	13925.962	0.845741285	0.848684652	0.212689142
	2	13608.468	13671.426	0.995394921		
	3	15572.054	13947.134	1.116505656		
	4	11739.75736	17225.962	0.681515341		
	5	9773.052382	16173.426	0.604266059		
UUO + PR-619	1	7060.933	16506.548	0.427765575	0.541823032	0.115939398
	2	7290.054	13144.841	0.554594308		
	3	9970.104	13667.548	0.729472763		
	4	7559.473767	14333.548	0.527397248		
	5	6889.795593	14662.719	0.469885264		

Figure 4A

		MMP2	GAPDH	MMP2/GAPDH	mean	SD
sham	1	7468.598	22739.678	0.328439039	0.382219896	0.049437647
	2	7219.841	16034.841	0.450259594		
	3	6015.255	16588.376	0.362618679		
	4	6365.962	17961.255	0.354427461		
	5	6413.426	15440.841	0.415354708		
UUO	1	17888.619	15406.426	1.161114135	1.078358002	0.13826453
	2	19939.134	15579.134	1.279861512		
	3	15574.255	15299.255	1.017974731		
	4	15363.376	16104.548	0.953977473		
	5	14488.962	14801.841	0.978862156		
UUO + PR-619	1	10172.669	12372.841	0.822177299	0.600671539	0.198310347
	2	10362.083	15012.134	0.690247169		
	3	10514.426	17039.426	0.617064565		
	4	4942.255	17357.548	0.284732325		
	5	11549.305	19603.79	0.589136335		

		MMP9	GAPDH	MMP9/GAPDH	mean	SD
sham	1	7712.134	22739.678	0.339148778	0.208987772	0.144286562
	2	6187.841	16034.841	0.385899742		
	3	2552.77	16588.376	0.153889085		
	4	1692.941	17961.255	0.094255162		
	5	1107.82	15440.841	0.071746092		
UUO	1	16913.012	15406.426	1.097789455	0.972659998	0.26066007
	2	16392.184	15579.134	1.052188395		
	3	19903.548	15299.255	1.300948837		
	4	11392.184	16104.548	0.707389242		
	5	10435.062	14801.841	0.704984062		
UUO + PR-619	1	8907.891	12372.841	0.719955182	0.588116953	0.238424344
	2	12190.891	15012.134	0.812069157		
	3	12716.184	17039.426	0.746280068		
	4	6392.477	17357.548	0.36828226		
	5	5763.477	19603.79	0.293998099		

Figure 5

		Smad2	GAPDH	Smad2/GAPDH	mean	SD
sham	1	12295.962	17422.255	0.705761797	0.465747782	0.23084214
	2	13340.669	18263.962	0.730436748		
	3	6037.719	20050.083	0.301131871		
	4	4993.548	15984.548	0.312398449		
	5	5612.719	20116.548	0.279010047		
UUO	1	21951.083	22785.205	0.963391947	1.017086914	0.256867202
	2	18313.548	18199.841	1.006247692		
	3	14374.548	17987.669	0.799133451		
	4	18616.376	12813.426	1.45288044		
	5	15024.426	17393.79	0.863781039		
UUO + PR-619	1	20522.083	19082.548	1.075437253	1.019893388	0.243005484
	2	14977.669	16911.134	0.88566911		
	3	15494.426	11796.012	1.313530878		
	4	8411.134	12325.134	0.68243753		
	5	18262.376	15986.083	1.142392167		

		Smad3	GAPDH	Smad3/GAPDH	mean	SD
sham	1	3067.305	17422.255	0.176056716	0.120018862	0.068057973
	2	3707.841	18263.962	0.203014056		
	3	1136.113	20050.083	0.056663755		
	4	1768.77	15984.548	0.11065499		
	5	1080.355	20116.548	0.053704791		
UUO	1	19516.79	22785.205	0.856555383	0.90516637	0.226608573
	2	15495.548	18199.841	0.851411174		
	3	13896.134	17987.669	0.772536675		
	4	16670.376	12813.426	1.301008489		
	5	12946.548	17393.79	0.744320128		
UUO + PR-619	1	18932.79	19082.548	0.992152096	0.660841897	0.265176181
	2	11336.548	16911.134	0.670360013		
	3	9545.962	11796.012	0.809253331		
	4	3627.719	12325.134	0.294335056		
	5	8602.255	15986.083	0.538108991		

		Smad4	GAPDH	Smad4/GAPDH	mean	SD
sham	1	7127.376	22739.678	0.313433462	0.421919255	0.091116626
	2	8773.376	16034.841	0.547144559		
	3	7741.426	16588.376	0.466677751		
	4	7574.255	17961.255	0.421699653		
	5	5568.598	15440.841	0.360640849		
UUO	1	16136.669	15406.426	1.047398598	1.057908377	0.153084062
	2	17463.548	15579.134	1.120957558		
	3	14115.719	15299.255	0.922640939		
	4	20671.376	16104.548	1.283573808		
	5	13543.255	14801.841	0.914970982		
UUO + PR-619	1	4505.426	12372.841	0.364138358	0.453714428	0.124035811
	2	8935.548	15012.134	0.595221705		
	3	8128.426	17039.426	0.477036374		
	4	5092.305	17357.548	0.293376979		
	5	10562.497	19603.79	0.538798722		

		TGF-βRI	GAPDH	TGF-βRI/GAPDH	mean	SD
sham	1	14221.456	23938.962	0.594071539	0.39706647	0.115397225
	2	9272.841	23640.669	0.392241057		
	3	5672.255	17500.841	0.324113281		
	4	7142.719	19352.083	0.369093033		
	5	5223.841	17081.79	0.305813442		
UUO	1	16283.962	12247.841	1.329537345	1.084958963	0.281298626
	2	12090.719	15698.426	0.770186705		
	3	12890.79	16000.962	0.805625937		
	4	20602.669	15194.719	1.355909839		
	5	18965.376	16299.79	1.16353499		
UUO + PR-619	1	14306.962	11163.134	1.28162593	1.16298192	0.192539801
	2	12694.962	11172.426	1.136276221		
	3	9738.962	10629.426	0.916226521		
	4	16376.376	11577.548	1.41449433		
	5	13587.962	12743.255	1.066286596		

		TGF-βRII	GAPDH	TGF-βRII/GAPDH	mean	SD
sham	1	17728.962	15524.426	1.142004349	1.040963759	0.144411441
	2	17842.548	22190.79	0.804051951		
	3	20699.255	17830.426	1.160895146		
	4	18395.548	16957.669	1.084792255		
	5	17911.426	17680.255	1.013075094		
UUO	1	10027.962	12761.012	0.785828115	0.497673209	0.251154351
	2	3939.305	16431.669	0.23973858		
	3	6879.719	16414.841	0.419115787		
	4	10965.012	14812.426	0.740257673		
	5	5180.305	17072.719	0.303425893		
UUO + PR-619	1	6313.012	16178.841	0.390201746	0.366482807	0.111218002
	2	3399.477	12469.134	0.272631363		
	3	3738.77	13202.012	0.283196985		
	4	7407.012	13550.134	0.546637546		
	5	4919.134	14478.841	0.339746393		

Figure 6

NRK-49F		α-SMA	β-actin	α-SMA/β-actin	mean	SD
Control	1	1104.79	21656.669	0.051013847	0.035354534	0.012125393
	2	523.234	22100.255	0.023675473		
	3	603.134	20156.962	0.02992187		
	4	902.134	19844.841	0.045459372		
	5	542.113	20302.255	0.026702108		
TGF-β1	1	19042.669	18731.255	1.016625368	0.979323721	0.060979751
	2	20065.548	20540.376	0.976883189		
	3	16533.548	18135.841	0.911650472		
	4	19647.134	18528.426	1.06037793		
	5	18259.134	19610.669	0.931081647		
TGF-β1 + PR-619	1	1170.305	18311.669	0.06391034	0.082041355	0.024324069
	2	2153.912	18748.255	0.114885999		
	3	1108.598	18778.255	0.059036263		
	4	1822.912	18183.669	0.100249955		
	5	1321.012	18315.79	0.072124216		

NRK-49F		Smad4	β-actin	Smad4/β-actin	mean	SD
Control	1	20810.497	20357.548	1.022249684	1.091290429	0.07131111
	2	20381.669	18675.548	1.091355873		
	3	20059.255	19494.962	1.028945581		
	4	20259.669	18124.548	1.117802717		
	5	19728.255	16493.841	1.196098289		
TGF-β1	1	17505.841	15690.962	1.115663973	1.071486524	0.043605332
	2	17843.669	16369.841	1.090033129		
	3	18767.548	17034.669	1.101726602		
	4	17241.719	16921.841	1.018903262		
	5	18912.376	18341.841	1.031105656		
TGF-β1 + PR-619	1	8320.841	12090.719	0.688200677	0.52535126	0.121546968
	2	8793.255	14815.426	0.593520227		
	3	7546.134	15328.255	0.492302222		
	4	7821.255	16075.548	0.486531159		
	5	6039.497	16492.255	0.366202014		
